# Patterns of Feline Coronavirus Shedding and Associated Factors in Cats from Breeding Catteries

**DOI:** 10.3390/v15061279

**Published:** 2023-05-30

**Authors:** Sandra Felten, Ute Klein-Richers, Stefan Unterer, Michèle Bergmann, Yury Zablotski, Regina Hofmann-Lehmann, Katrin Hartmann

**Affiliations:** 1 Clinic of Small Animal Medicine, Centre for Clinical Veterinary Medicine, LMU Munich, Veterinärstrasse 13, 80539 Munich, Germany; uteklein1981@gmail.com (U.K.-R.); n.bergmann@medizinische-kleintierklinik.de (M.B.); y.zablotski@med.vetmed.uni-muenchen.de (Y.Z.); hartmann@lmu.de (K.H.); 2 Clinic for Small Animal Internal Medicine, Department for Small Animals, Vetsuisse Faculty, University of Zurich, Winterthurerstrasse 260, 8057 Zurich, Switzerland; stefan.unterer@uzh.ch; 3 Clinical Laboratory, Department of Clinical Diagnostics and Services, and Center for Clinical Studies, Vetsuisse Faculty, University of Zurich, Winterthurerstrasse 260, 8057 Zurich, Switzerland; rhofmann@vetclinics.uzh.ch

**Keywords:** FCoV, feces, carrier, intermittent shedding, feline enteric coronavirus, FECV, feline infectious peritonitis, FIP, RT-PCR

## Abstract

(1) Background: In households in which feline coronavirus (FCoV) is present, three patterns of FCoV shedding are described: non-shedders, intermittent (low-intensity) shedders, or persistent (high-intensity) shedders. It was the aim of this study to describe FCoV shedding patterns in cats from catteries in which FCoV infection is endemic. Additionally, risk factors for high-intensity FCoV shedding or non-shedding were analyzed. (2) Methods: Four fecal samples of 222 purebred cats from 37 breeding catteries were examined for FCoV RNA by quantitative reverse transcription polymerase chain reaction (RT-qPCR). High-intensity shedders were defined as cats positive for FCoV RNA in at least 3/4 fecal samples; non-shedding cats were defined as cats negative in all four fecal samples. Risk factor analysis was performed using information obtained by questionnaire. (3) Results: Of the 222 cats, 125 (56.3%) were considered high-intensity shedders, while 54/222 cats (24.3%) were FCoV non-shedders. The Persian breed was associated with a higher risk of high-intensity shedding in multivariable analysis, while Birman and Norwegian Forest Cats were more likely to be FCoV non-shedders. Cats living together with other cats were more likely to be FCoV shedders. (4) Conclusions: The proportion of both high-intensity shedders and non-shedding cats was higher than previously reported, which possibly can be explained by housing conditions, different genetic susceptibility, or differences in the study period. The risk of high-intensity shedding is higher in certain breeds. However, it cannot be excluded that the individual hygiene procedure of each breeder influenced FCoV-shedding frequency. A smaller group size is a protective factor against FCoV shedding.

## 1. Introduction

Feline coronavirus (FCoV) infection is widespread among domestic felids worldwide. Shedding prevalence is especially high in multi-cat environments, in which cats live in close contact, share litter boxes, as well as sleeping and feeding spaces [1]. FCoV shedders are present in most catteries and in shelters with more than five cats [1,2]. FCoV is a single-stranded, enveloped RNA virus belonging to the genus *Alphacoronavirus* within the family *Coronaviridae* [3]. It is assumed that the virus can preserve infectivity for days to a few weeks [4] and thus can be transmitted efficiently directly or indirectly via the fecal-oral route [5]. After infection, FCoV replicates preferentially within the apical columnar epithelium of the duodenum, jejunum, ileum, and colon [6]. Viral replication in chronic FCoV infection is mainly located in the colon and rectum [6]. Infection can be associated with mild diarrhea but remains mostly without clinical signs [5,7]. Infected cats are thought to shed FCoV in three possible patterns: cats can shed FCoV intermittently, either due to true intermittent shedding or recovery and re-infection; some cats shed FCoV for a given time and then clear the infection and stop shedding; and some cats can become (even lifelong) carriers persistently shedding FCoV in their feces [5,8,9,10,11]. It is known that young cats between one and five years of age are more likely to shed FCoV [1] and also to shed higher amounts of FCoV [5] compared to older cats. Additionally, immunosuppression (e.g., due to feline immunodeficiency virus or feline leukemia virus infection) can cause shedding of higher viral loads [5,12] and longer shedding periods [12], whereas pregnancy, parturition, lactation [5,10], or methylprednisolone acetate treatment had no influence on FCoV shedding [5]. However, it has not been extensively studied which potential risk factors (e.g., signalment, housing conditions, and management techniques) are associated with either of the described FCoV shedding patterns; although, it would be crucial to know which risk factors might be associated with chronic and high-intensity shedding for epidemiologic and management purposes in multi-cat environments. This is important especially because FCoV infection is the prerequisite to the development of feline infectious peritonitis (FIP), a severe systemic multi-inflammatory syndrome that occurs in up to 12% of FCoV-infected cats in multi-cat environments [13]. FIP develops after mutation of FCoV within an infected cat, leading to a change in viral cell tropism and enabling efficient viral replication in macrophages [12,14]. The number of cats shedding FCoV within a multi-cat environment was identified as a risk factor for the development of FIP in individual cats in these households [15]. 

Natural resistance to FIP has been linked to both viral characteristics and host immune response, and the role of genetics in FIP resistance has been studied by some research groups [16,17,18,19,20,21,22,23,24,25]. Conclusive evidence to link FIP to certain changes in the cats’ genome, however, is missing. Purebred cats in general and certain breeds seem more susceptible to the development of FIP [16,17,18,19]. Some cats living in multi-cat environments with endemic FCoV infection are not infected with FCoV and do not shed the virus despite a high risk of infection (“non-shedders” or “resistant cats”). These cats have not been more intensively studied and characterized so far. 

Therefore, it was the aim of this study to describe patterns of FCoV shedding in cats from breeding catteries housing at least five cats and to describe a potential association of factors (e.g., signalment, housing conditions, and management techniques) with the observed FCoV shedding patterns. In addition, cats not shedding FCoV should be described in more detail.

## 2. Materials and Methods

### 2.1. Cats and Catteries

This prospective study included 222 cats from 37 breeding catteries. These catteries all kept at least five cats and at least one intact queen for breeding. Catteries were distributed all over Germany and regions near the border of Germany. Breeders were contacted either via telephone, email, or personally at cat shows. Seventy-six of the 222 cats were male (60 sexually intact, 16 neutered) and 146 were female (130 sexually intact, 16 neutered). The age of the cats ranged from 2 months to 15.5 years; 51 cats were <1 year of age, 135 cats were 1–5 years of age, and 34 cats were >5 years of age. The age of two cats was not recorded. Of the 222 cats, 58 were from catteries housing groups of 5–10 cats and 164 were from catteries housing >10 cats. All cats were purebred; breeds included British Shorthair (BSH; *n* = 55; from 10 different catteries), Bengal (*n* = 51; from 8 different catteries), Birman (*n* = 30; from 4 different catteries), Persian (*n* = 18; from 1 cattery), Norwegian Forest Cat (*n* = 16; from 2 different catteries), Maine Coon (*n* = 16; from 6 different catteries), Somali (*n* = 9; from 3 different catteries), Turkish Van (*n* = 7; from 1 cattery), Scottish Fold (*n* = 4; from 2 different catteries), Scottish Straight (*n* = 4; from 1 cattery), Sphynx (*n* = 4; from 1 cattery), Turkish Angora (*n* = 3; from 1 cattery), Savannah (*n* = 1), Oriental (*n* = 1), and Taiga (*n* = 1). The breed of two cats was not recorded. Of the 37 catteries, six kept more than one breed of cats (Bengal and Taiga, Bengal and Sphynx, Bengal and Savannah, Scottish Fold and Scottish Straight, BSH and Scottish Fold, and Turkish Van and Maine Coon, respectively). Breeders were free to sample as many cats from their cattery as they desired. As a consequence, not all cats from each cattery were included in the study.

Some of the cats were part of previous studies [1,7]. Samples were collected from February 2016 until November 2017. The animal study protocol was approved by the responsible veterinary authority (Regierung von Oberbayern; reference number 55.2-1-54-2532.2-14-13) and cat breeders gave their informed consent prior to participation.

### 2.2. Samples

Breeders collected four consecutive fecal samples from each cat. Breeders were instructed to collect the fecal samples at time intervals of 5–28 days. The first three fecal samples from each cat were immediately frozen and kept at −18 °C until analysis. The fourth fecal sample of each cat was analyzed within 48 h of defecation and kept at 4 °C until analysis. 

### 2.3. Reverse Transcription Polymerase Chain Reaction (RT-PCR)

Quantitative RT-PCR (RT-qPCR) was used to detect FCoV RNA in fecal samples as described previously [26] at a commercial laboratory (IDEXX Laboratories, Kornwestheim, Germany). For this, total nucleic acid was extracted from fecal samples by applying the “MagVet™ Universal Kit” (ThermoFisher, Darmstadt, Germany) on a KingFisher Flex Purification System platform (ThermoFisher, Darmstadt, Germany) according to the manufacturer’s instructions. RT-qPCR based on the FCoV 7b gene [26] was performed as a singleplex reaction. RT-qPCR was run with six quality controls, including RT-qPCR-positive controls, RT-qPCR-negative controls, negative extraction controls, an internal positive control (IPC) spiked into the lysis solution to monitor the nucleic acid extraction efficiency, and the presence or absence of inhibitory substances, RNA quality control, and an environmental contamination monitoring control [27]. Samples with a cycle threshold (Ct) value below 40 were considered positive. If RT-qPCR was initially weakly positive (Ct ≥ 40), RT-qPCR was repeated in duplicate. Further interpretation of the RT-qPCR was as follows: samples with one Ct value <40 and one Ct value ≥40 were considered positive. Samples with two Ct values ≥40 were considered weak positive (low concentration of FCoV RNA detected). Samples with only one Ct value ≥40 and the other one negative were considered below the limit of quantification. Samples without a Ct value in both analyses were considered negative. Weak positive samples were considered positive for further analysis (but had to be excluded from the analysis of mean fecal FCoV load since no FCoV load could be determined). Samples below the limit of quantification were considered negative for further analysis. Cats with only one weak RT-qPCR-positive or one below the limit of quantification and three RT-qPCR-negative fecal samples each were excluded from all analyses involving fecal FCoV load since mean fecal FCoV load could not be determined. In all other cats, the mean fecal FCoV load of all four fecal samples was calculated and shown in viral copies per gram (g) of feces.

### 2.4. Risk Factor Analysis

All cat breeders filled in a questionnaire covering 12 potential categorical (sex, breed, frequency of litter box cleaning per day, contact with other cats outside the cattery, outdoor access, and feeding of raw meat) or continuous (age, number of cats living together in the cattery, overall space in the cattery, available space per cat, ratio of cats and litter boxes, and frequency of litter box disinfection per month) risk factors including the cats’ signalment, as well as housing and management factors. Additionally, the breeder was analyzed as an individual risk factor.

### 2.5. Statistical Analysis

Cats were considered high-intensity shedders if they shed FCoV RNA in at least three of the four fecal samples analyzed. The normality of distribution of the mean FCoV load/g feces in both groups (low- and high-intensity shedders) was evaluated via the Shapiro-Wilk normality test. Due to a not-normal distribution of data, the Mann-Whitney U test was applied in order to analyze the association between mean FCoV load/g feces and the frequency of FCoV shedding. 

Proportions of high-intensity FCoV shedders (positive in ≥3 fecal samples) and proportions of non-shedding cats (negative in all 4 fecal samples) for every potential risk factor were studied with logistic regressions. First, univariable logistic regressions were applied to all risk factors in order to determine factors that could potentially be associated with the pattern of FCoV shedding. As recommended by Dohoo et al. [28], in cases where the *p*-value of the univariable analysis was ≤0.2, the risk factor was suspected to have an association with high-intensity shedding or to be a protective factor against FCoV shedding and was considered for the multivariable logistic regression. Backwards stepwise elimination via Akaike’s information criterion was then applied in order to (1) control for confounding factors and (2) reduce the number of variables to only potentially influential ones while (3), at the same time, maximizing model quality. The assumption of multicollinearity in the multivariable model was assessed by the variance inflation factors (VIF) Only VIF <5 were accepted in the final model. For breed as a risk factor, breeds represented by <5 cats were combined into one mixed group “<5 cats”). 

Data analysis was performed using R version 4.0.3 (10 October 2020, R Foundation for Statistical Computing, Vienna, Austria). Results with a *p*-value < 0.05 in multivariable analysis were considered statistically significant.

## 3. Results

### 3.1. Patterns of FCoV Shedding

Of the 222 cats, 125 (56.3%) shed FCoV RNA in at least three samples and, thus, were considered high-intensity shedders. Almost all of those cats (111/125; 88.8%) shed FCoV RNA in all four fecal samples. On the other hand, only 43/222 (19.4%) cats shed FCoV intermittently and, thus, were considered low-intensity shedders. In contrast, 54/222 (24.3%) of the cats did not shed FCoV RNA in any of the four fecal samples. 

**Figure 1:**
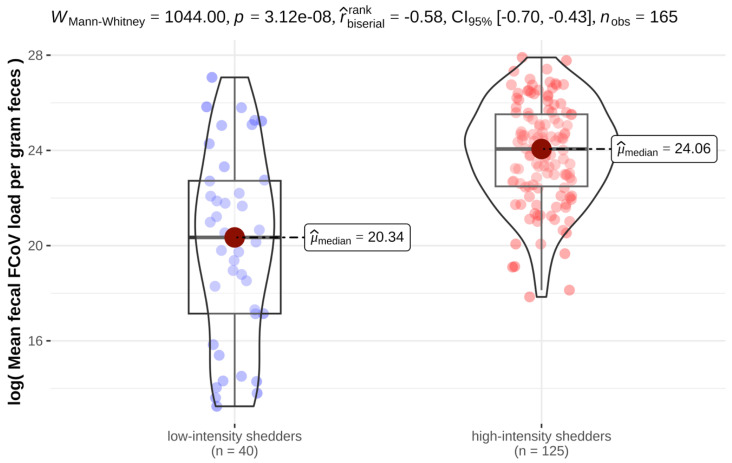
Violin plots depicting the significant correlation (*p* < 0.001) between mean feline coronavirus (FCoV) loads per gram (g) of feces (log of mean FCoV load) between low-intensity and high-intensity shedders. Three cats had to be excluded from the analysis because all of their RT-qPCR-positive samples yielded weak positive results, precluding analysis of fecal FCoV load.

The mean fecal FCoV load calculated from four fecal samples per cat in all cats ranged from 5.68 × 10^5^ to 1.32 × 10^12^ viral copies/g feces (mean 9.37 × 10^10^ viral copies/g feces). Three cats with intermittent shedding had to be excluded from the analysis of mean fecal FCoV load because their RT-qPCR-positive samples only yielded weak positive results, and thus, fecal FCoV load could not be determined in these samples. There was a significant correlation between mean FCoV load/g feces and the frequency of FCoV shedding: high-intensity shedders (cats shedding FCoV in at least three of the four fecal samples) also shed higher amounts of FCoV (Figure 1).

### 3.2. Risk Factor Analysis

**Figure 2:**
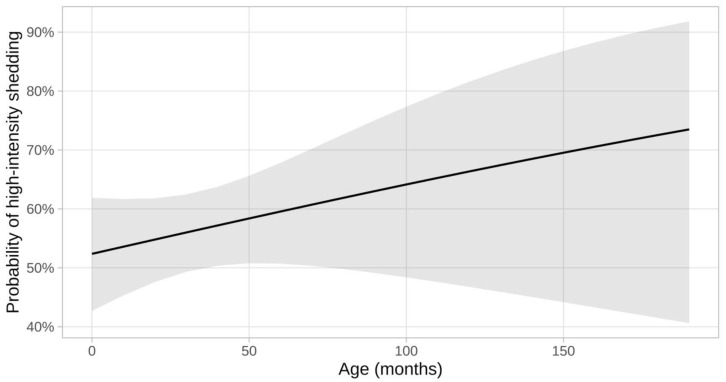
Probability of high-intensity shedding at different ages (in months). The grey shading indicates 95% confidence intervals. There was no significant association between age and high-intensity shedding in univariable analysis (*p* = 0.262) and, therefore, age did not become part of the multivariable model.

Six of the 12 potential risk factors analyzed (breed, number of cats living together in the cattery, frequency of litter box cleaning per day, frequency of litter box disinfection per month, outdoor access, and feeding of raw meat) were shown to potentially be associated with an increased risk for being a high-intensity shedder according to the univariable analysis. In the multivariable model, however, only breed and the frequency of litter box cleaning per day were significantly associated with high-intensity shedding (Table 1 and Table 2). The age of the cats was not significantly associated with high-intensity shedding according to univariable analysis (Figure 2). Since the individual breeder and the breed of the cats were associated with a VIF > 5, multicollinearity was presumed and the factor breeder was eliminated from the final model.

**Figure 3:**
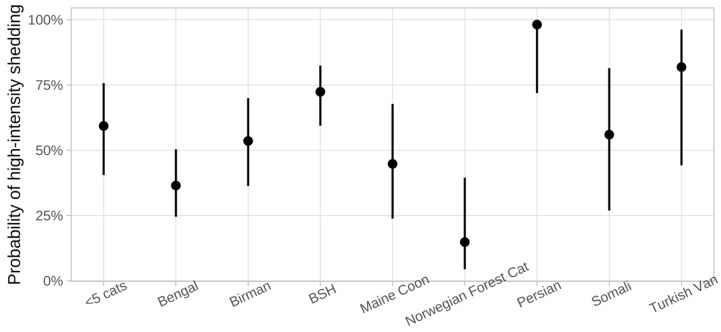
Probability of high-intensity shedding in the different breeds included in the study. Breeds were only depicted separately if at least 5 cats of the breed were included. Norwegian Forest Cats (*p* = 0.026, odds ratio (OR) 0.17, 95% confidence interval (CI) 0.04–0.82) had a lower risk; Persians had a higher risk of being a high-intensity shedder (*p* = 0.031, OR 28.98, 95%, CI 1.33–630.79). Dots represent the medians; bars represent 95% confidence intervals. Breeds represented by <5 cats were combined into one mixed group (<5 cats). BSH = British Shorthair.

Norwegian Forest Cats (*p* = 0.026, OR 0.17, 95% CI 0.04–0.82) had a lower risk of being a high-intensity shedder compared to cats of the other breeds, whereas Persians had a higher risk of being a high-intensity shedder compared to cats of the other breeds (*p* = 0.031, OR 28.98, 95% CI 1.33–630.79). However, confidence intervals were wide (Figure 3). A factor negatively associated with high-intensity shedding was the frequency of litter box cleaning per day (*p* = 0.018, OR 1.06, 95% CI 1.01–1.10): cats from catteries in which litter boxes were cleaned more often had a higher risk of being a high-intensity shedder (Table 1 and Table 2).

### 3.3. Non-Shedding Cats

When characterizing the 54 non-shedding cats, 7 factors were shown to potentially be a protective factor for FCoV shedding according to the univariable analysis: breed (*p* < 0.001), outdoor access (*p* = 0.192), the number of cats living together in the cattery (*p* = 0.012), the ratio of cats and litter boxes (*p* = 0.063), the frequency of litter box cleaning per day (*p* = 0.012), the frequency of litter box disinfection per month (*p* = 0.059), and contact with other cats outside the cattery (*p* = 0.043). After multivariable analysis, however, only breed, the number of cats living together in the cattery, and the frequency of litter box disinfection per month were significantly associated with not shedding (Table 3 and Table 4).

Birman (*p* = 0.003, OR 7.55, 95% CI 1.95–29.19) and Norwegian Forest Cats (*p* = 0.003, OR 8.70, 95% CI 2.07–36.51) were significantly more often FCoV non-shedders compared to the other breeds. However, confidence intervals were wide. Cats living together in a cattery with a smaller number of cats (*p* = 0.006, OR 0.91, 95% CI 0.85–0.97) and cats from catteries in which litter boxes were disinfected less often (*p* = 0.026, OR 0.81, 95% CI 0.68–0.98) also were more likely to be non-shedders (Table 3 and Table 4).

## 4. Discussion

This study aimed to describe patterns of FCoV shedding in cats from catteries in which FCoV infection was endemic. For this, four fecal samples were analyzed per cat, and cats were categorized accordingly: cats shedding FCoV in three of the four analyzed samples, equating to shedding ≥75% of the time, were defined as high-intensity shedders. All other cats that shed FCoV in one or two fecal samples were defined as low-intensity shedders. Cats that in none of the four fecal samples shed FCoV were considered non-shedders and potentially resistant to FCoV infection. Potential risk factors associated with high-intensity shedding and factors associated with non-shedding of FCoV were evaluated.

The majority of cats (75.7%) in the present study shed FCoV at least once. This reflects the high prevalence of FCoV infection known to occur in multi-cat environments [1,11,29]. Nevertheless, the percentage of cats that did not shed FCoV during the whole study period (24.3%) was substantially higher than what has been shown in previous studies, in which only 2.9–9.1% of cats did not shed FCoV [10,11]. The reason for this higher percentage of non-shedding cats is unknown. However, the previously performed studies are rather old (from the years 1997 and 2001) and it is possible that breeders were not as informed about accurate hygiene measures as breeders are today. However, hygiene and husbandry measures were not significantly associated with high-intensity shedding or non-shedding in the multivariable analysis. FCoV shedding can be intermittent. Still, it is unlikely that intermittent shedding was missed in the present study. Fecal samples were collected at intervals of 5–28 days. This is in accordance with the current recommendations for fecal sampling [30,31] that fecal samples should be collected at intervals of one week to one month for analysis by RT-qPCR in order not to underestimate the number of intermittently shedding cats [1]. Some previous studies have even collected fecal samples at longer time intervals of up to three months [5,9,10,11], which makes it even more unlikely that FCoV-shedding was underestimated in the present study. Another possible explanation for a higher proportion of FCoV-negative cats is the inclusion of less susceptible cats. It is possible that the present study included cat lineages with different genetic susceptibility or resistance to FCoV infection compared with other studies. In general, the susceptibility to FCoV infection and/or the development of FIP might be influenced by both viral factors and host immune response [20,21,32]. Purebred cats are thought to be more susceptible to the development of FIP [16,17,19,33]. On the other hand, no specific breed was identified as a risk factor for FCoV infection in healthy cats in Germany [1]. In the present study, Norwegian Forest Cats and Birmans had a lower risk to be FCoV-infected, which might be explained by a “resistant” genotype. A few studies examined a possible relationship between heritable genetic host factors and an increased incidence of FIP and found single nucleotide polymorphisms (SNPs) in the feline interferon-gamma gene to be associated with the outcome of FCoV infection [23,24,25]. However, small numbers sometimes limited statistical analysis in those studies [25], and there was some overlap between cats with and without FIP, indicating that identifying the SNPs is of limited value in individual cats and, therefore, unfortunately, cannot be used to guide breeding [24].

It is, of course, also not known whether the non-shedding cats in the present study were truly resistant to FCoV infection or had been infected and shedding before and eventually stopped fecal FCoV shedding. To evaluate this, long-term follow-up studies would be necessary.

Looking at shedding frequency in the FCoV-shedding cats, the majority (56.3%) were high-intensity shedders with fecal samples positive for FCoV RNA ≥75% of the time. Only 19.4% of the cats shed FCoV intermittently. These proportions differ from what has been reported in the literature. One study by Foley et al., applying similar categorization criteria as the present study, followed 275 cats from catteries for one year at sampling intervals of one to three months and found the majority of cats (53.7%) shed FCoV with moderate frequency (25–75% of the time), whereas only 8.3% of the cats shed FCoV with high frequency (>75% of the time) [10]. Another study by Harpold et al., examining 33 cats from an Abyssinian cattery with endemic FCoV infection for 13 months at sampling intervals of two months, found 26.7% of the cats shed FCoV >75% of the time [9]. Other studies did not categorize cats based on the frequency of FCoV shedding in sequential fecal samples, making direct comparisons difficult [5,8,11]. Nevertheless, it becomes clear that most previous studies found much higher proportions (32.4–82.6%) of cats shedding FCoV intermittently [5,9,10,11]. Addie et al. collected samples from 155 cats from 29 households at intervals of at least one month and found 13.2% of the cats shed FCoV persistently and 32.4% shed intermittently; 41.2% shed FCoV for a limited time and then ceased shedding, and 2.9% never shed FCoV [11]. Pedersen et al. found equal proportions of persistently (33.3%) and intermittently shedding cats (36.4%) when following 33 experimentally FCoV-infected cats [5]. 

When characterizing high-intensity shedding cats in the present study even further, it could be shown that, in total, 111 of the 125 high-intensity shedders (89%) and, thus, 111/222 (50%) of the whole study population shed FCoV for the entire study period. These cats likely have persistent FCoV infections. Previous studies have only found 13.2–33.3% of the cats to be persistent FCoV shedders [5,9,10,11]. Nevertheless, since fecal samples were collected at different sampling intervals of 5–28 days in the present study, it cannot be excluded that some of the cats with four FCoV RNA-positive samples cleared the infection after the study period, and thus were not long-term persistent shedders.

One possible explanation for these discrepancies in shedding-pattern frequency might be the different housing conditions of the cats in the various studies. In the present study, only cats from catteries housing at least five cats were included. In fact, most of the cats (164/222) were from catteries that housed >10 cats. In some other studies, cats were housed together with no more than five cats per room [5] or even single-cat households were included [11]. As demonstrated in the risk factor analysis in the present study, cats not shedding FCoV were less frequently encountered in households in which more cats lived in close contact and shared litter boxes and feeding places, likely leading to constant exposure to infectious material. Since immunity is short-lasting, this high infectious pressure can lead to an increased risk of spread and persistence of FCoV within a household [32]. It is also possible that some of the cats were genetically more susceptible to chronic FCoV infection. It is likely that viral factors, such as the infecting dose [34], and host factors, such as host immunity [32], play a role in the course of FCoV infection. A distinct genotype might be associated with the persistence of FCoV infection and one study has identified an association between certain candidate gene markers potentially involved in innate immunity and the phenotype of persistent/high-intensity FCoV shedding [35]. As shown in the multivariable risk factor analysis in the present study, Persian cats had a higher risk of being high-intensity shedders, and in fact, all Persian cats included were high-intensity shedders. All these cats were from the same breeder and were living in one of two individual groups of 12 or 16 cats. On the other hand, Norwegian Forest Cats significantly less often shed FCoV with high frequency. Only two Norwegian Forest Cats were high-intensity shedders; these two cats were from two distinct catteries housing 8 and 20 cats, respectively. Another possible explanation for the higher percentage of persistently FCoV-infected cats in the present study might be a distinctive sampling procedure. In the present study, only voided fecal samples were analyzed for FCoV RNA by RT-qPCR. Previous studies sometimes used fecal swabs instead of voided samples [5,10,11]. It is known that FCoV RNA loads are significantly lower in fecal swabs compared to voided fecal samples [36]. Consequently, it cannot be excluded that shedding episodes might have been missed in studies using fecal swabs for RNA detection. Finally, it is possible that the RT-qPCR assay used for FCoV RNA detection in the present study was more sensitive than the assays used in previous studies, thereby, detecting a higher proportion of cats (persistently) shedding FCoV.

Previous studies mostly followed cats for a longer time and categorized cats that were initially shedding FCoV and then ceased shedding after some months as a separate entity [5,9,10,11]. A study involving laboratory cats demonstrated that fecal FCoV shedding remained high after experimental FCoV infection for 2–10 months before evolving into the 3 shedding patterns: intermittent shedding, persistent shedding, and recovery from shedding [5]. From the results of the present study, it is not possible to determine the proportion of FCoV-infected cats that eventually would cease shedding since cats were only followed for a period of up to four months. Thus, it cannot be excluded that some of the cats persistently shedding FCoV later recovered from shedding or started to shed FCoV only intermittently.

In the present study, cats were categorized as high-intensity shedders based on shedding frequency rather than fecal FCoV RNA loads. Still, knowing the amount of FCoV RNA that is shed in the feces of a cat is important in order to estimate the odds of infecting other cats. To the authors’ knowledge, however, there is no evidence for distinct cut-offs of fecal virus loads that can routinely be used to distinguish between high- and low-intensity shedders. In order to determine whether higher FCoV-shedding frequency in this population of cats also correlated with a higher fecal FCoV RNA load, the mean fecal FCoV load was calculated from all four fecal samples per cat in all cats. Indeed, high-intensity shedders also shed higher amounts of FCoV. This is in accordance with previous reports [37,38]. Nevertheless, since a cut-off of fecal FCoV load for high-intensity shedding cannot be defined from the present data, the examination of multiple fecal samples by RT-qPCR is still necessary in order to detect high-intensity FCoV shedders.

Multivariable risk factor analysis suggested that the breed and the frequency of litter box cleaning per day were associated with high-intensity shedding. Accordingly, breed, the number of cats living together in the cattery, and the frequency of litter box disinfection per month were associated with the non-shedding of FCoV. Even more factors were associated with high-intensity FCoV shedding or non-shedding in univariable analysis, but most of them were eliminated from the final model. It is likely that the individual hygiene procedures of each breeder influenced FCoV-shedding frequency, thereby distorting the results of the univariable analysis. It could be demonstrated before that high-frequency shedding was associated with the cattery of origin, likely reflecting individual husbandry and hygiene management and, possibly, heritable differences [10]. In the present study, multicollinearity was detected between the individual breeder and the breed of the cats; therefore, the breeder as a risk factor was eliminated from the final model.

As mentioned before, the breed of the cats was associated with a higher or lower risk of high-intensity FCoV shedding or non-shedding in this population of cats. This could potentially be explained by different genetic susceptibility. Nevertheless, confidence intervals were quite wide, indicating that further studies including larger numbers of cats per breed are necessary to truly confirm the effect of the breed on the likelihood of high-intensity shedding or resistance to FCoV infection. It cannot be excluded that the breeders’ (which mostly kept only one, and rarely two, different breeds in their cattery) effect on husbandry and hygiene influenced the results. 

The frequency of litter box cleaning per day was negatively associated with high-intensity shedding, meaning that the risk of high-intensity shedding increased with higher frequency of litter box cleaning. Additionally, the probability that a cat was a non-shedder decreased with a higher frequency of litter box disinfection. These results do not follow logical aspects. Of course, one would expect the risk of high-intensity shedding to increase with less litter box cleaning since this would increase the contact time of cats with the highly infectious FCoV. Likewise, one would expect more cats to be FCoV RNA-negative in an environment in which litter boxes are disinfected more frequently. One potential explanation could be that high-intensity shedders more frequently had diarrhea and thus, breeders more regularly cleaned and disinfected litter boxes in groups with high-intensity shedders (as it is known that FCoV infection can be associated with diarrhea [7]). However, such linkage has not been evaluated in the present study. Longitudinal analysis of fecal samples by a fecal scoring system would have been required to further investigate this potential correlation. Ultimately, the reason for these discordant results in the present study is unknown. Since risk factor analysis had to be performed based on information given by the cat breeders, this could not be verified and distortion of the results cannot be excluded.

Age was not associated with high-intensity shedding in the present study. This is rather unexpected since previous studies have suggested young age to not only be a risk factor for FCoV-shedding in general [1] but also for high-frequency shedding [10] and shedding of higher FCoV RNA loads [5]. 

This study had a few limitations. First, risk factor analysis was carried out based on the questionnaire the cat breeders completed. Thus, the information given was subjective and could not be proven. Additionally, there might be some selection bias since breeders were not selected using a standardized approach and the decision to participate was voluntary. Consequently, there might have been a bias towards committed breeders. Since breeders could decide how many of their cats they wished to include in the study, it could be argued that this could also have influenced the results. Nevertheless, FCoV shedding prevalence was comparable to that reported before, and, thus, selection bias was considered neglectable. Further, cats were only followed for a period of up to four months. In future studies, it would be interesting to analyze fecal samples at regular intervals for longer periods in order to verify the shedding-pattern frequency and associated risk factors. 

## 5. Conclusions

This study analyzing FCoV shedding patterns in cats from breeding catteries with endemic FCoV infection found higher proportions of both high-intensity FCoV shedding and cats not shedding FCoV than previously reported. Possibly, this can be explained by differences in housing and hygiene management. The Persian breed was a risk factor for high-intensity shedding, while Birman and Norwegian Forest Cats were more likely to be FCoV non-shedders. Reducing the number of cats living together in groups remains the most important step in FCoV infection control. This study also might provide implications for coronavirus epidemiology in other species, including severe acute respiratory syndrome coronavirus type 2.

## Figures and Tables

**Table 1:** Evaluated categorical risk factors and their influence on high-intensity feline coronavirus (FCoV) shedding in univariable and multivariable analyses. Individual breeds were only included separately in the analysis if more than five cats were represented. Other breeds were combined into one mixed group (<5 cats). Results presented in bold were statistically significant in either univariable (*p* ≤ 0.2) or multivariable (*p* < 0.05) analysis.

						Multivariable Analysis	
Risk Factor	Possible Categories (Number of Cats)	Number of High-Intensity Shedding Cats (*n* = 125)	Number of Low-Intensity Shedding Cats (*n* = 43)	Number of Cats without Shedding (*n* = 54)	Uni- Variable Analysis *p*-Value	*p*-Value	Odds Ratio (95% CI)
Sex	Female intact (*n* = 130)	73 (56.1%)	27 (20.8%)	30 (23.1%)	0.307	-	-
	Female neutered (*n* = 16)	10 (62.5%)	1 (6.3%)	5 (31.3%)			
	Male intact (*n* = 60)	30 (50.0%)	13 (21.7%)	17 (56.7%)			
	Male neutered (*n* = 16)	12 (75.0%)	2 (12.5%)	2 (12.5%)			
	<5 cats (*n* = 20)	12 (60.0%)	5 (25.0%)	3 (15.0%)		reference	reference
Breed	BSH (*n* = 55)	40 (72.7%)	9 (16.4%)	6 (10.9%)	**<0.001**	0.116	2.28 (0.81-6.44)
	Bengal (*n* = 51)	19 (37.3%)	16 (31.4%)	16 (31.4%)		0.11	0.46 (0.17–1.20)
	Birman (*n* = 30)	16 (53.3%)	3 (10.0%)	11 (36.7%)		0.286	0.53 (0.17–1.70)
	NFC (*n* = 16)	2 (12.5%)	4 (25.0%)	10 (62.5%)		**0.026**	**0.17 (0.04–0.82)**
	Persian (*n* = 18)	18 (100%)	0	0		**0.031**	**28.98 (1.33–630.79)**
	Maine Coon (*n* = 16)	7 (43.8%)	3 (18.8%)	6 (37.5%)		0.482	0.65 (0.19–2.20)
	Somali (*n* = 9)	5 (55.6%)	2 (22.2%)	2 (22.2%)		0.369	2.03 (0.43–9.55)
	Turkish Van (*n* = 7)	6 (85.7%)	1 (14.3%)	0		0.352	2.46 (0.37–16.54)
Cleaning of litter boxes per day	<2 times (*n* = 63)	25 (39.7%)	16 (25.4%)	22 (34.9%)	**0.006**	reference	reference
	2–3 times (*n* = 113)	69 (61.1%)	17 (15.0%)	27 (23.9%)		**0.014**	**2.66 (1.21–5.83)**
	>3 times (*n* = 46)	31 (67.4%)	10 (21.7%)	5 (10.9%)		**0.026**	**2.88 (1.13–7.36)**
Contact with other cats	Yes (*n* = 167)	90 (53.9%)	31 (18.6%)	46 (27.5%)	0.204	-	-
	No (*n* = 55)	35 (63.6%)	12 (21.8%)	8 (14.5%)			
Outdoor access	Yes (*n* = 94)	47 (50.0%)	20 (21.3%)	27 (28.7%)	**0.105**	n.s.	n.s.
	No (*n* = 128)	78 (60.9%)	23 (18.0%)	27 (21.1%)			
Feeding of raw meat	Yes (*n* = 167)	86 (51.5%)	40 (24.0%)	41 (24.6%)	**0.011**	n.s.	n.s.
	No (*n* = 55)	39 (70.9%)	3 (5.5%)	13 (23.6%)			

*n* = number of cats; BSH = British Shorthair; NFC = Norwegian Forest Cat; n.s. = not significant; 95% CI = 95% confidence interval.

**Table 2:** Evaluated continuous risk factors and their influence on high-intensity feline coronavirus (FCoV) shedding in univariable and multivariable analyses. Results presented in bold were statistically significant in either univariable (*p* ≤ 0.2) or multivariable (*p* < 0.05) analysis.

					Multivariable Analysis	
Risk Factor	Median in High-Intensity Shedding Cats (*n* = 125)	Median in Low-Intensity Shedding Cats (*n* = 43)	Median in Cats without Shedding (*n* = 54)	Uni- Variable Analysis *p*-Value	*p*-Value	Odds Ratio (95% CI)
Age (months)	26	20	24	0.262	-	-
Number of cats in a cattery	15	15	13	**0.093**	n.s.	n.s.
Space in total (m^2^)	165	165	130	0.629	-	-
Space per cat (m^2^)	10.8	10.8	10.5	0.489	-	-
Ratio of cats and litter boxes	1.7	1.7	1.6	0.220	-	-
Frequency of litter box disinfection per month	4	2	2	**0.132**	n.s.	n.s.

*n* = number of cats; n.s. = not significant; 95% CI = 95% confidence interval.

**Table 3:** Evaluated categorical risk factors and their influence on the non-shedding of feline coronavirus (FCoV) in univariable and multivariable analyses. Individual breeds were only included separately in the analysis if more than five cats were represented. Other breeds were combined into one mixed group (<5 cats). Results presented in bold were statistically significant in either univariable (*p* ≤ 0.2) or multivariable (*p* < 0.05) analysis.

					Multivariable Analysis	
Risk Factor	Possible Categories (Number of Cats)	Number of Shedders (*n* = 168)	Number of Non-Shedders (*n* = 54)	Uni- Variable Analysis *p*-Value	*p*-Value	Odds Ratio (95% CI)
Sex	Female intact (*n* = 130)	100 (76.9%)	30 (23.1%)	0.495	-	-
	Female neutered (*n* = 16)	11 (68.8%)	5 (31.3%)			
	Male intact (*n* = 60)	43 (71.2%)	17 (56.7%)			
	Male neutered (*n* = 16)	14 (87.5%)	2 (12.5%)			
Breed	<5 cats (*n* = 20)	17 (85.0%)	3 (15.0%)	**<0.001**	reference	reference
	BSH (*n* = 55)	49 (89.1%)	6 (10.9%)		0.291	0.50 (0.14–1.83)
	Bengal (*n* = 51)	35 (68.6%)	16 (31.4%)		0.076	2.87 (0.89–9.29)
	Birman (*n* = 30)	19 (63.3%)	11 (36.7%)		**0.003**	**7.55 (1.95–29.19)**
	NFC (*n* = 16)	6 (37.5%)	10 (62.5%)		**0.003**	**8.70 (2.07–36.51)**
	Persian (*n* = 18)	18 (100%)	0		0.239	0.17 (0.01–3.27)
	Maine Coon (*n* = 16)	10 (62.5%)	6 (37.5%)		0.159	2.66 (0.68–10.46)
	Somali (*n* = 9)	7 (77.8%)	2 (22.2%)		0.519	0.57 (0.10–3.14)
	Turkish Van (*n* = 7)	7 (100%)	0		0.42	0.29 (0.01–6.07)
Cleaning of litter boxes per day	<2 times (*n* = 63)	41 (65.1%)	22 (34.9%)	**0.012**	n.s.	n.s.
	2–3 times (*n* = 113)	86 (76.1%)	27 (23.9%)			
	>3 times (*n* = 46)	41 (89.1%)	5 (10.9%)			
Contact with other cats	Yes (*n* = 167)	121 (72.5%)	46 (27.5%)	**0.043**	n.s.	n.s.
	No (*n* = 55)	47 (85.5%)	8 (14.5%)			
Outdoor access	Yes (*n* = 94)	67 (71.3%)	27 (28.7%)	**0.192**	n.s.	n.s.
	No (*n* = 128)	101 (78.9%)	27 (21.1%)			
Feeding of raw meat	Yes (*n* = 167)	126 (75.4%)	41 (24.6%)	0.891	-	-
	No (*n* = 55)	42 (76.4%)	13 (23.6%)			

*n* = number of cats; BSH = British Shorthair; NFC = Norwegian Forest Cat; n.s. = not significant; 95% CI = 95% confidence interval.

**Table 4:** Evaluated continuous risk factors and their influence on the non-shedding of feline coronavirus (FCoV) in univariable and multivariable analyses. Results presented in bold were statistically significant in either univariable (*p* ≤ 0.2) or multivariable (*p* < 0.05) analysis.

				Multivariable Analysis	
Risk Factor	Median in Shedders (*n* = 168)	Median in Non-Shedders (*n* = 54)	Uni- Variable Analysis *p*-Value	*p*-Value	Odds Ratio (95% CI)
Age (months)	22	24	0.595	-	-
Number of cats in a cattery	15	13	**0.012**	**0.006**	**0.91 (0.85–0.97)**
Space in total (m^2^)	165	130	0.889	-	-
Space per cat (m^2^)	10.8	10.5	0.216	-	-
Ratio of cats and litter boxes	1.7	1.6	**0.063**	n.s.	n.s.
Frequency of litter box disinfection per month	4	2	**0.059**	**0.026**	**0.81 (0.68–0.98)**

*n* = number of cats; n.s. = not significant; 95% CI = 95% confidence interval.

## Data Availability

Not applicable.
